# Detection of incorrect manufacturer labelling of hip components

**DOI:** 10.1007/s00256-016-2478-4

**Published:** 2016-09-22

**Authors:** Matthieu Durand-Hill, Johann Henckel, Matthew Burwell, John Skinner, Alister Hart

**Affiliations:** 1 Institute of Orthopaedics, University College London, London, UK; 2 Royal United Hospital, Bath, UK

**Keywords:** Computed tomography, X-ray, Arthroplasty, Replacement, Hip, Prostheses and implants, Postoperative care, Hip prosthesis

## Abstract

We describe the case of a 53-year-old man who underwent a left metal-on-metal hip resurfacing in 2015. Component size mismatch (CSM) was suspected because of the patient’s immediate post-operative mechanical symptoms and high metal ion levels. Surgical notes indicated the appropriate combinations of implants were used. However, we detected a mismatch using computed tomography. Revision was performed and subsequent measurements of explanted components confirmed the mismatch. To our knowledge, this case is the first report of a CT method being used in a patient to pre-operatively identify CSM.

**Electronic supplementary material:**

The online version of this article (doi:10.1007/s00256-016-2478-4) contains supplementary material, which is available to authorized users

## Introduction

Size mismatch of components used in total hip arthroplasty, is a “serious, largely preventable” incident that has devastating effects for patients [1]. The risk of component size mismatch (CSM) is predicted to rise, owing to growing numbers of increasingly complex components being used in orthopaedics [2]. The National Joint Registry for England, Wales and Northern Ireland reported that 62 out of the 9,676 revisions performed in 2013 were due to CSM of the head–acetabular socket (0.64 %) [3]. However, it is likely that CSM is under-estimated (with an incidence closer to 1 %), because of difficulties in intra-operative and plain radiograph detection [2].

**Fig. 1:**
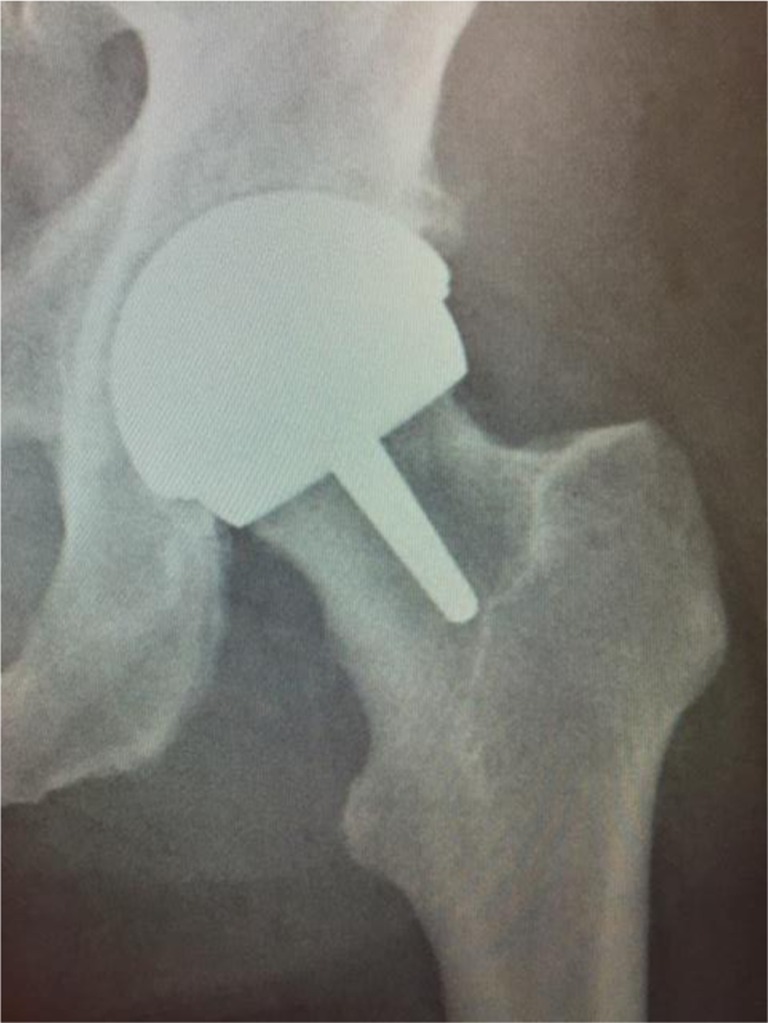
Post-operative anterior–posterior plain film radiograph of the patient’s left hip. No cause of the patient’s mechanical symptoms was detected

**Fig. 2:**
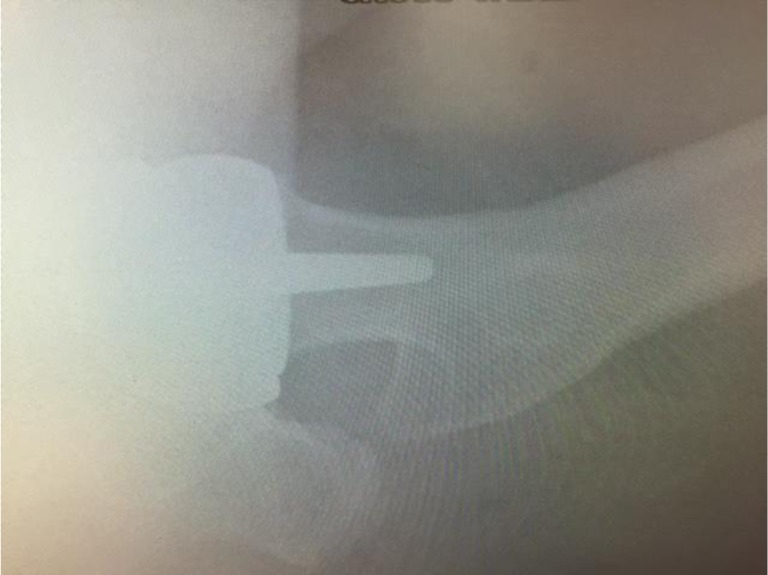
Post-operative lateral plain film radiograph of the patient’s left hip. No cause of the patient’s mechanical symptoms was detected

The detection of metal-on-metal (MoM) CSM using plain radiographs remains challenging (Figs. 1, 2). In a recent survey, the mean detection rate, by surgeons using plain radiographs, of MoM CSM was 27.7 % (see Appendix) [2]. The low contrast between the cup and head component in MoM hip replacements, combined with the low intended clearance of bearing surfaces (approximately 200 μm) explains the difficulty in radiograph detection. In contrast, detection of metal-on-polyethylene CSM is possible using plain radiographs, and has been reported previously [4, 5].

To our knowledge, this case is the first report of CT being used in a patient to pre-operatively identify CSM. This manuscript does not address the medico-legal implications of this issue.

## Case report

In 2015, a 53-year-old man (with previous right hip resurfacing) underwent a MoM hip resurfacing for left hip osteoarthritis. Intra-operatively, the consultant orthopaedic surgeon (who has 17 years’ experience performing total hip resurfacing) suspected that the head was undersized compared with the acetabular cup. The component’s engravings could not be visualised intra-operatively. The implant manufacturer was informed via the representative, but reported that the size of the components would be as marked on the boxes. Hence, the components were left in situ. The acetabular cup’s outer diameter was recorded (using the manufacturer’s labels) as 56 mm, the acetabular bearing surface diameter was 48 mm and the femoral head component’s diameter was recorded as 48 mm.

Anterior–posterior and lateral X-rays of the patient’s hip were acquired (Figs. 1, 2). No obvious abnormalities were seen on the radiographs and the patient was subsequently discharged with an early 2-week review, because of the surgeon’s initial intra-operative suspicion of CSM. At 2 weeks the patient’s incision had healed and the patient reported mechanical symptoms of instability and “squelching” of the component. This was very dissimilar to the immediate post-operative symptoms of the previous contralateral hip resurfacing.

Blood cobalt and chromium levels were measured at 5 weeks post-operatively. Five-week metal ion results were significantly elevated: cobalt was 69.3 ppb and chromium was 54.8 ppb. This, combined with the patient’s mechanical post-operative symptoms, apparently normal AP and lateral radiographs and the initial suspicion of CSM, prompted further imaging.

**Fig. 3:**
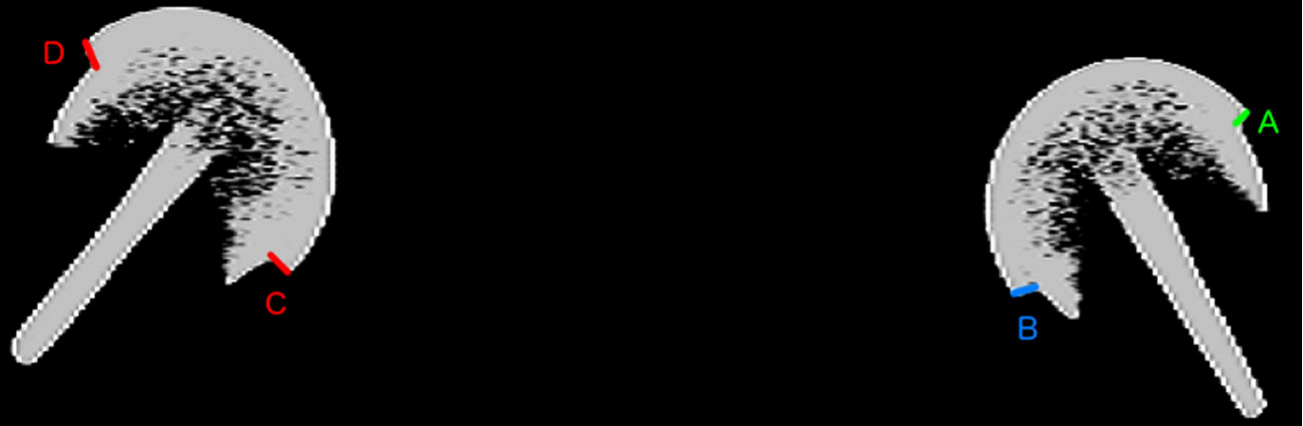
A 2D CT coronal slice of the patient with the mismatched left hip resurfacing (*right*) and the patient’s previous right hip resurfacing (*left*) in view. The femoral head of the mismatched component is not sitting centrally in the acetabular component with* B* being 1.5 times greater than* A*. On the previous right hip resurfacing, with correctly paired components, the femoral head is centrally placed (*C* to* D*)

**Fig. 4:**
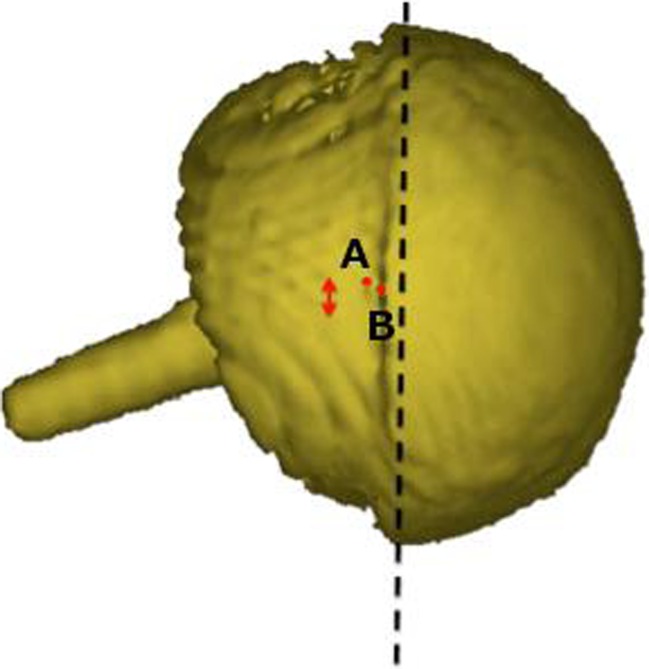
Three-dimensional model of the patient’s metal-on-metal (MoM) resurfacing. The acetabular (*A*) and femoral head (*B*) centres of rotation have been marked and the* red arrow* demonstrates that the two points are not overlapping, which indicated component size mismatch (CSM)

**Fig. 5:**
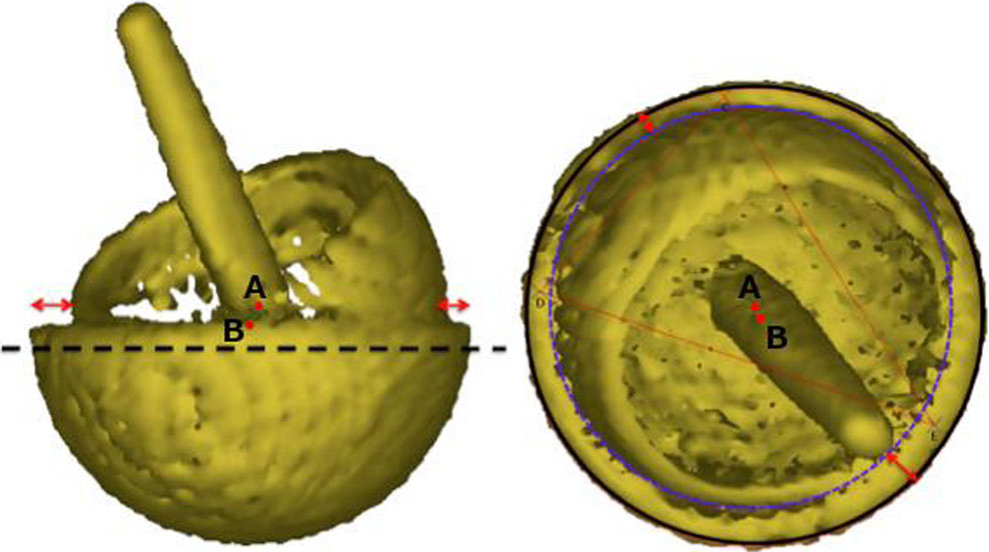
Lateral (*left*) and true anterior–posterior of the acetabular cup (*right*) views of a three-dimensional model of the patient’s MoM resurfacing.* A* is the acetabular centre of rotation and* B* is the femoral head centre of rotation. The* red arrows* indicate that the femoral head is not centred in the acetabulum, which suggests CSM

Consequently, a low-dose CT scan (1.4 mSv) of the patient’s hip was obtained (Fig. 3). The low-dose CT scan was obtained using a SOMATOM Definition AS 128-slice CT scanner (Siemens Heathineers, Erlangen, Germany) using the CT protocol shown in Table 1. Three-dimensional models of the components were produced. The centre of rotation of each component was compared (Fig. 4) and the distance between the edge of the acetabular cup and femoral head surface was measured (Fig. 5). This method works on the premise that a fully engaged head should be equally spaced from the outer surface of the acetabular cup and that the centre of rotation of spheres fitted to the outer surface of the acetabular and femoral head components should overlap, provided that an offset-bore acetabular component has not been used.

**Table 1:** Parameters of the low-dose CT protocol

Area scanned	kV	mAs	Scan length (cm)^a^	Collimation
ASIS–stem tip	100	100	30	128*0.625
Knee	100	70	20	128*0.625
Ankle	100	45	10	24*1.25

*ASIS* anterior superior iliac spine

^a^Scan length varies depending upon patient size and component. An anterior–posterior topogram should be acquired to determine scan range. All areas should be scanned using a pitch of 1.0 and rotation time of 1.0 s and reconstructed using 1-mm slices and the kernel “BONE”. An extended scale should be used.

Three-dimensional analyses of the CT scan indicated a potential CSM (Figs. 4, 5). Results suggested that the acetabular-bearing diameter was 50 mm, highly suggestive of a 2-mm CSM, with the acetabular bearing surface 2 mm greater than the femoral head. Revision was recommended on this basis.

The company was informed throughout the post-operative follow-up via the local representative. Based on the information that the company had seen they advised against revision surgery and suggested that close clinical follow-up of the patient might be arranged.

**Fig. 6:**
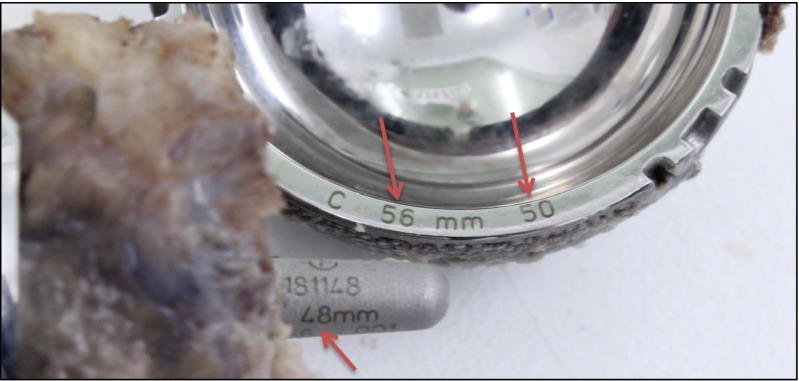
Photo of the revised Adept hip resurfacing removed from the 53-year-old gentleman. Manufacturer labels in the surgical notes record the components as being a 56-mm cup designed for a 48-mm head and a 48-mm head. Engravings (*red arrows*) and coordinate measuring machine measurements confirm the CSM, as the cup is actually a 56-mm cup designed for a 50-mm head

The resurfacing was revised 10 weeks post-primary surgery, with the Adept hip resurfacing (MatOrtho, Surrey, UK) exchanged for a Trinity Metafix (Corin, Cirencester, UK). The explanted Adept hip resurfacing components, both acetabular and femoral components, were sent to the London Implant Retrieval Centre for analysis. On visual inspection, implant labelling pointed to a bearing size mismatch (Fig. 6). These measurements were confirmed using a coordinate measuring machine (internal diameter of the cup = 49.98 mm, outer diameter of the head = 47.84 mm). Wear analysis of both components was also performed. The volume of wear in the acetabular component was 20.59 mm^3^. This gives a predicted 1-year wear of 107 mm^3^. The volume of wear of the femoral head component was 10.14 mm^3^. This gives a predicted 1-year wear of 47.84 mm^3^. Under standard walking conditions, with correctly positioned components, assuming 1.9 million cycles per annum [6], the expected wear rate should be less than 2 mm^3^/year [7].

## Discussion

Detection of MoM CSM is very challenging using plain radiographs [2]. However, we have developed a method that uses CT to identify CSM in MoM patients. Our imaging technique includes: 3D CT with metal artefact reduction, a low radiation dose protocol and a software measurement technique [8]. The main limitation of the technique is the requirement to know component specifics regarding dimensions (e.g. if there is a designed offset bore [9]). This poses a problem if the types of component implanted/design specifics of the component are unknown. Another limitation of the technique is that it is time-consuming and requires proficiency in 3D CT analysis software. This may limit its use in clinical practice.

**Fig. 7:**
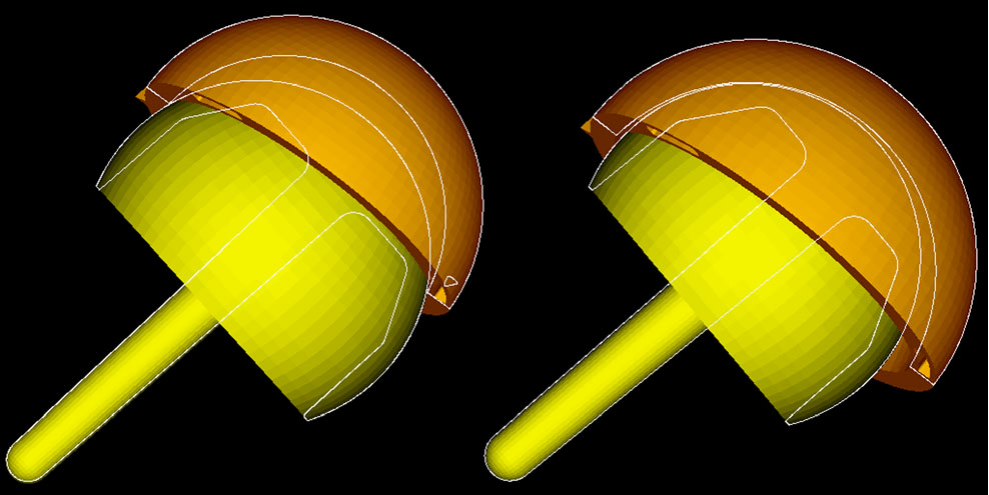
Two 3D models of potential CSMs: oversized femoral head (*left*); oversized acetabulum (*right*). The* white outline* reveals that the femoral head sits centrally in the acetabulum in cases where the femoral head is oversized. In CSM cases where the acetabulum is oversized (as presented) the femoral head does not sit centrally

The same principle can be applied to 2D CT slices (Fig. 3), by comparing the distance between the outer surface of the acetabulum and the femoral head (AC–FH). Owing to metal artefact this was only possible at the edge of the acetabulum. For this reason, patients with mismatches due to oversized heads may go undetected because at the acetabular edge oversized femoral heads are centrally located and the AC–FH distance is equal (Fig. 7). We therefore recommend that 3D models are still generated to accurately visualise the difference between the femoral and acetabular centre of rotations in all planes.

The method was used to detect a mislabelled oversized cup, as the centre of rotation of the acetabular and femoral head components did not overlap, plus the femoral head did not sit centrally in the acetabular component (Figs. 4, 5). If the femoral component was oversized, the centres of rotation of the acetabulum and femoral head components would not overlap, but the femoral head would remain centrally positioned in the acetabulum (Fig. 7).

Metal ions are known to increase in patients who have MoM implants and there is increasing concern over their toxicity [10]. Furthermore, there is no clear consensus on what counts as normal chromium and cobalt ion levels. The best published evidence has suggested that the normal upper limit of blood metal ions in patients with MoM implants is between 0.5 and 2.5 ppb for chromium and 0.7 and 3.4 ppb for cobalt [11]. Another study indicated serum chromium ion levels above 17 ppb and serum cobalt levels above 19 ppb are likely to result in metallosis [12]. In this case, the patient had metal ions far higher than the normal limit in MoM patients. In addition, the patient would likely have developed metallosis had the detection of CSM and prompt revision not occurred [12].

**Fig. 8:**
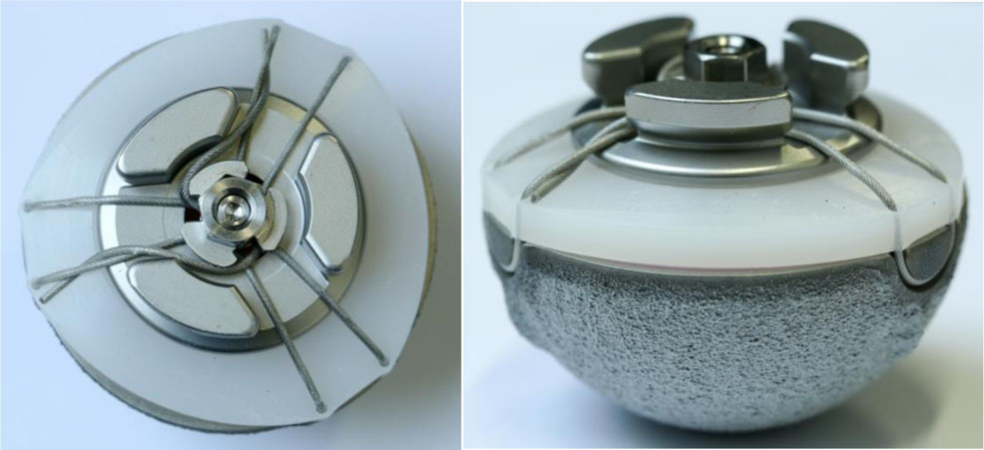
Photo of an acetabular component with the impactor cap still attached. The impactor cap covers the engravings and is removed only after insertion

Currently, proper procedure dictates that only the labels on the boxes are checked and noted. This is because, while the engravings on the acetabulum (Fig. 6) can be seen at revision, they are not visible before implantation as they are packaged with an impactor cap attached (Fig. 8).

Mislabelling of components has previously been reported on two occasions. In both instances the manufacturer recalled the device, a statement was made by the US Food and Drug Administration and a Medical Device Alert was given by the Medicines and Healthcare Regulatory Agency [13, 14]. Advice from the Medicines and Healthcare Regulatory Agency suggested that the plain radiographs of anyone suspected of being fitted with an incorrect prosthesis should be obtained [14]. However, previous work has demonstrated that CSM may be missed using plain radiographs [2].

Cases of mismatch due to surgical error (mismatching of correctly packaged components) have also previously been reported. However, these cases do not require imaging for detection, provided that the notes are available to be checked.

To reduce the incidence of CSM, we recommend that component sizes are documented on the whiteboard in theatre (in the same way the swab count check is performed). In addition, this case highlights the importance of attempting to check the engravings on the acetabular component after insertion and not just relying on the boxes/stickers. However, this is not always achievable as the implant lies deep in the wound, the engravings are often obscured by blood or overhanging acetabular bone and the engravings may not be on the same side as the surgeon. Furthermore, even if the surgeon saw “56 mm”, there are two different acetabular components with an external diameter of 56 mm. The external diameter of 56 mm is also one of two acceptable pairings that can be used with a 48-mm head. Consequently, both numbers need to be visualised.

Future work will incorporate the lessons learned from this case and retrieval data on mismatches to develop a modified WHO checklist with the aim of reducing the incidence of CSM. Within the modified WHO checklist it would be imperative for the engravings on the components to be directly visualised, either post-implantation or, if this proves too difficult to implement, pre-implantation, following a redesign of the impactor caps.

We applied our method to a patient referred with suspected CSM. Based on our results we recommended revision surgery, and analysis of explanted components confirmed the CSM. To our knowledge, this case is the first report of a CT method being used in a patient to pre-operatively identify CSM. We believe that this CT method will be useful in identifying CSM of MoM components in patients whose surgical notes are unobtainable.

## Electronic supplementary material

Below is the link to the electronic supplementary material.

ESM 1 (DOCX 6648 kb)
