# Supplementary material for: Detection of incorrect manufacturer labelling of hip components
Source: Skeletal Radiol. 2016 Sep 22;46(1):105–9. doi: 10.1007/s00256-016-2478-4 (PMC5121176; doi:10.1007/s00256-016-2478-4)
Supplement: Supplementary file 1 — (DOCX 6648 kb) [file 256_2016_2478_MOESM1_ESM.docx]

**Appendix**

**
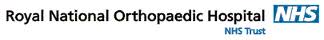
**

**
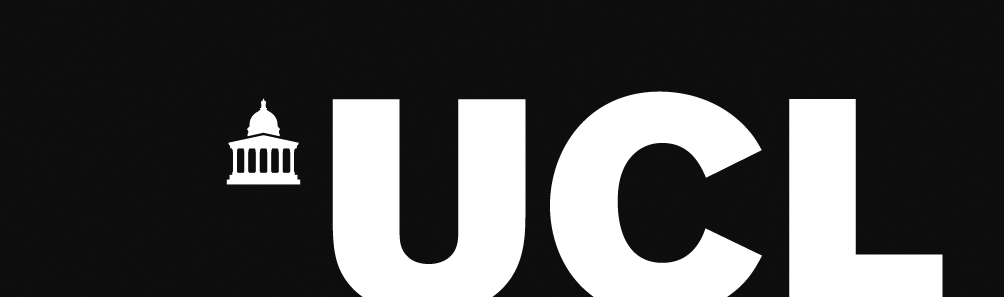
**

**Metal on Metal Hip Radiographs Questionnaire**

**Please look at each radiograph individually and comment on the pathology noted. (using the box to the right of each image).**

**Please ensure to comment regarding the**

- **Bones**
- **Soft Tissues**
- **Components (implant type, size and position)**

| LIRC No. | **X-Ray** | **Comments** |
| --- | --- | --- |
| 019-JM | 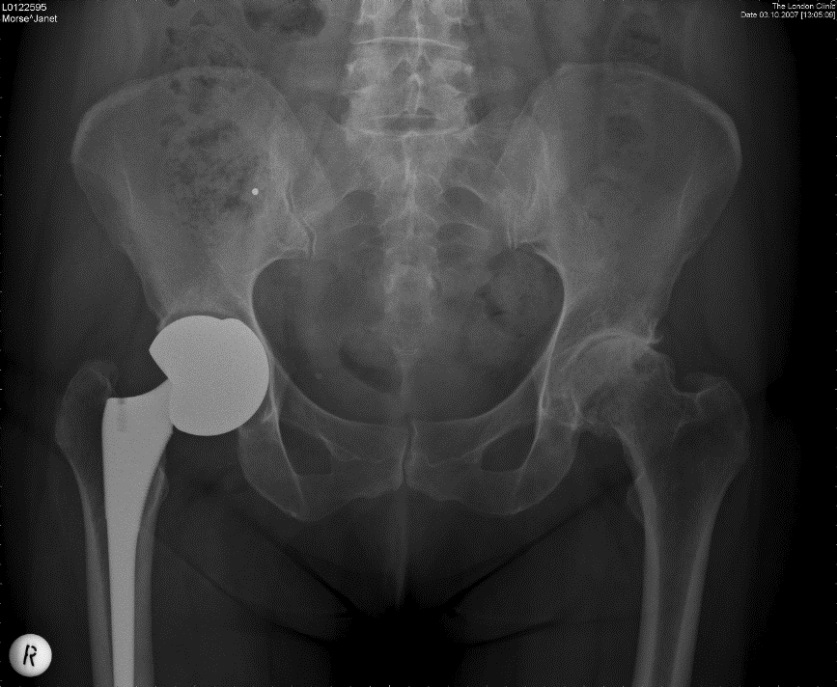 |  |
| 070-RM | 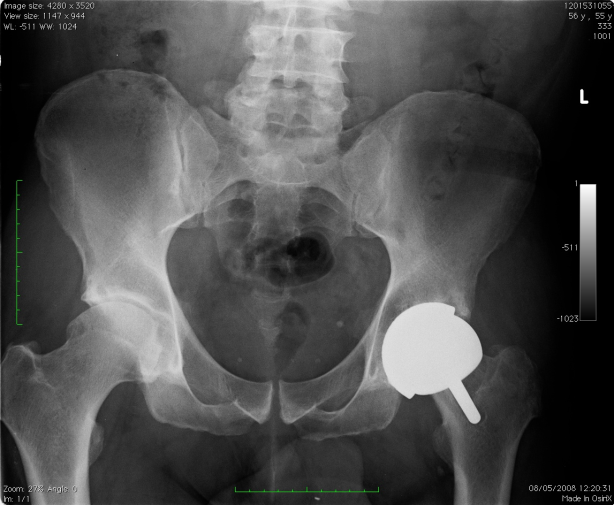 |  |
| 075-DL | 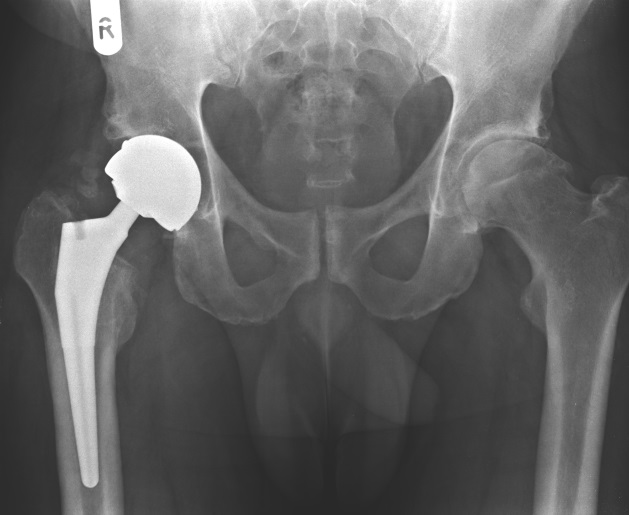 |  |
| 107-KT | 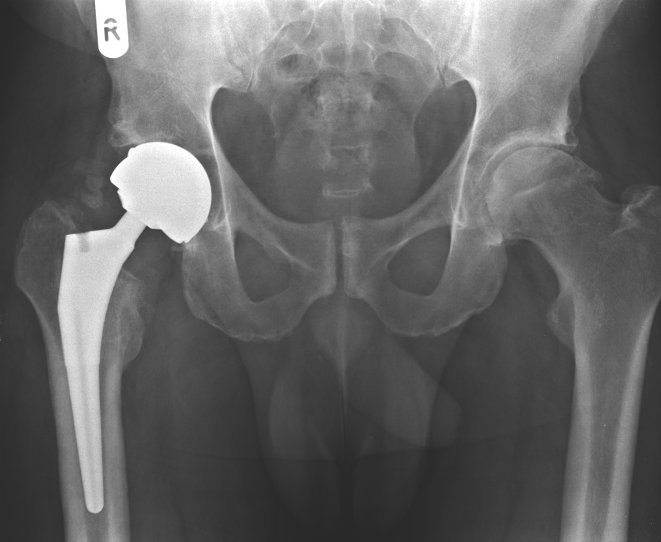 |  |
| 121-TC | 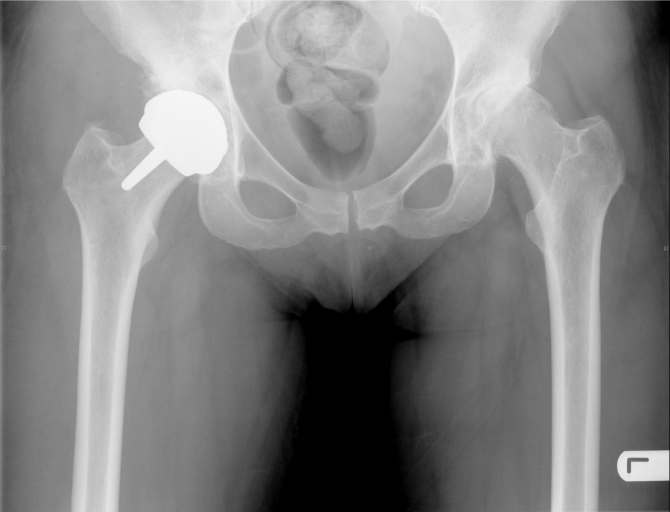 |  |
| 152-PA | 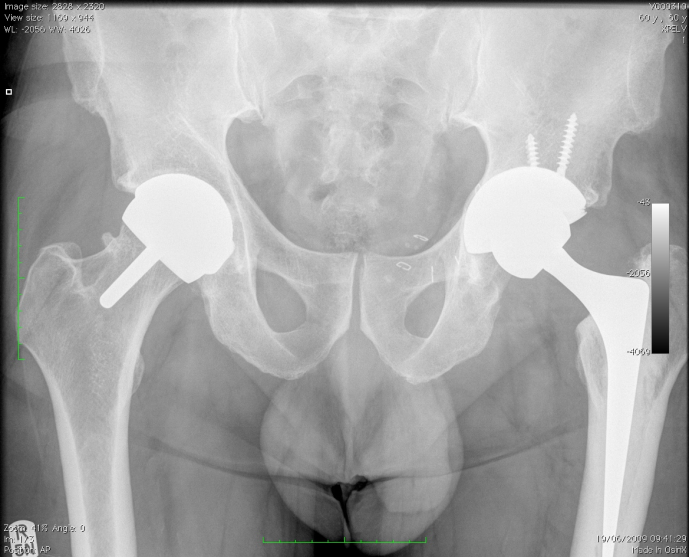 | (Please refer to Left Hip) |
| 176-DW | 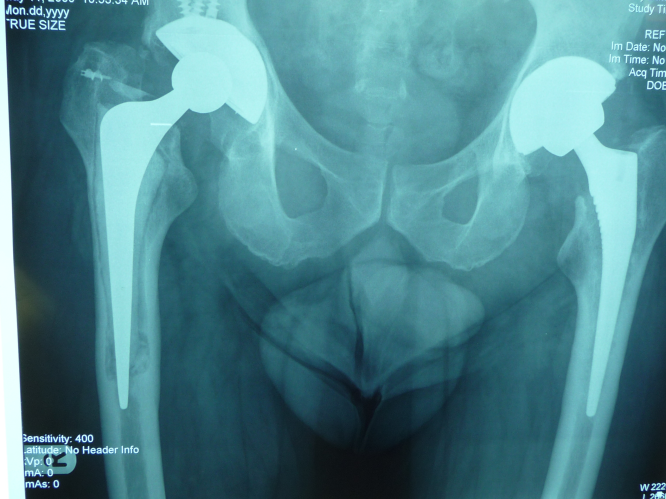 | (Please refer to Left Hip) |
| 229-DP | 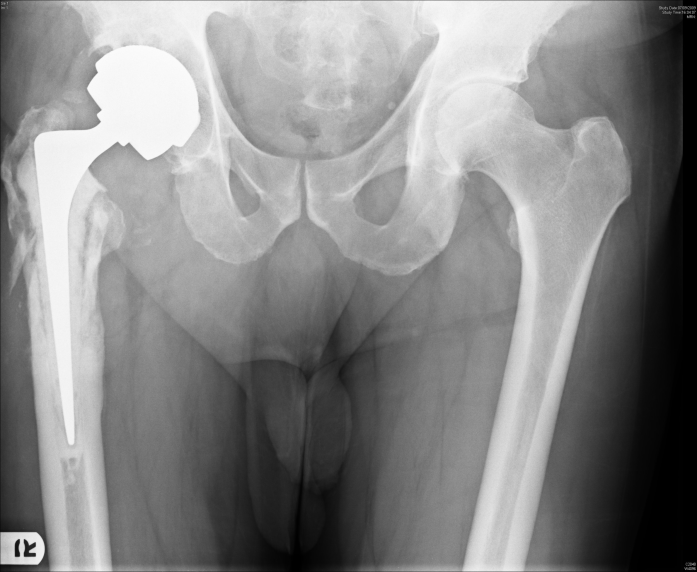 |  |
| 287-JS | 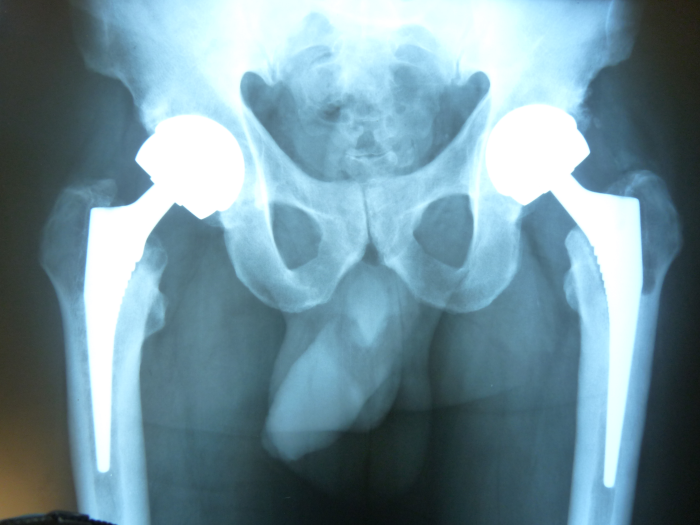 | (Please refer to Left Hip) |
| 310-FF | 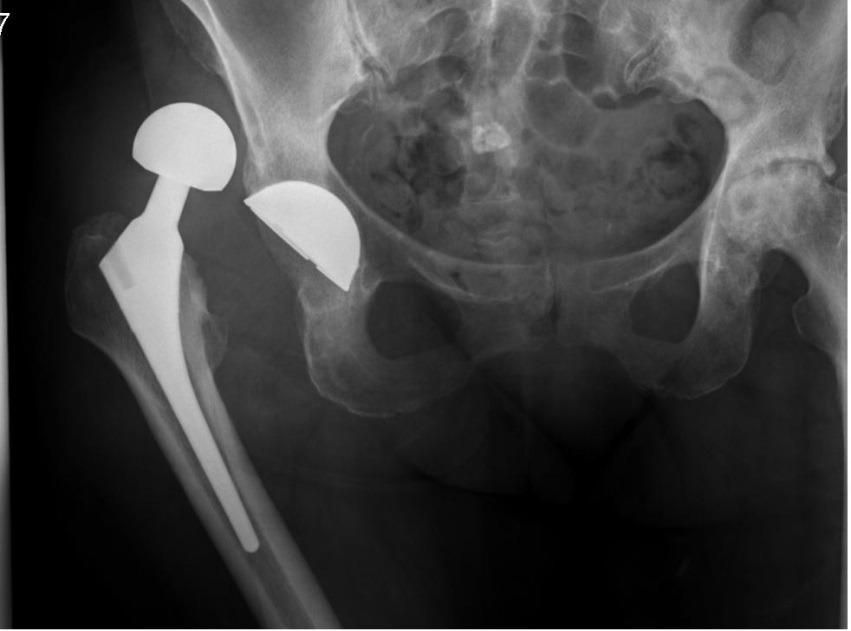 |  |
| 311-AK | 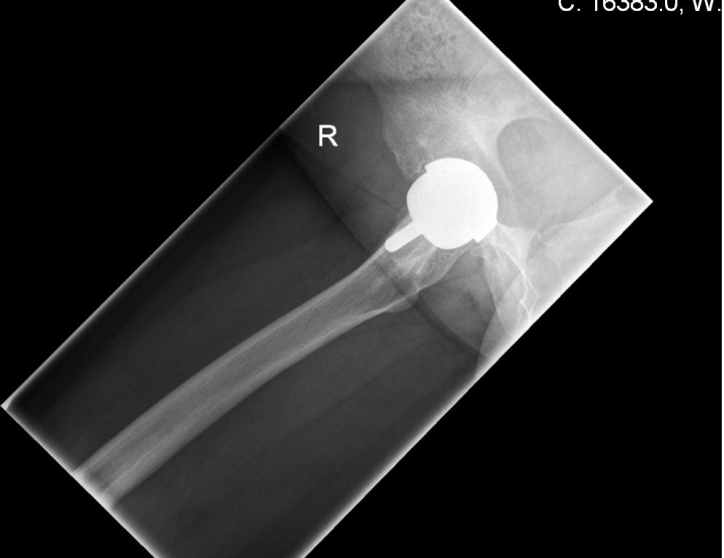 |  |
| 435-MB | 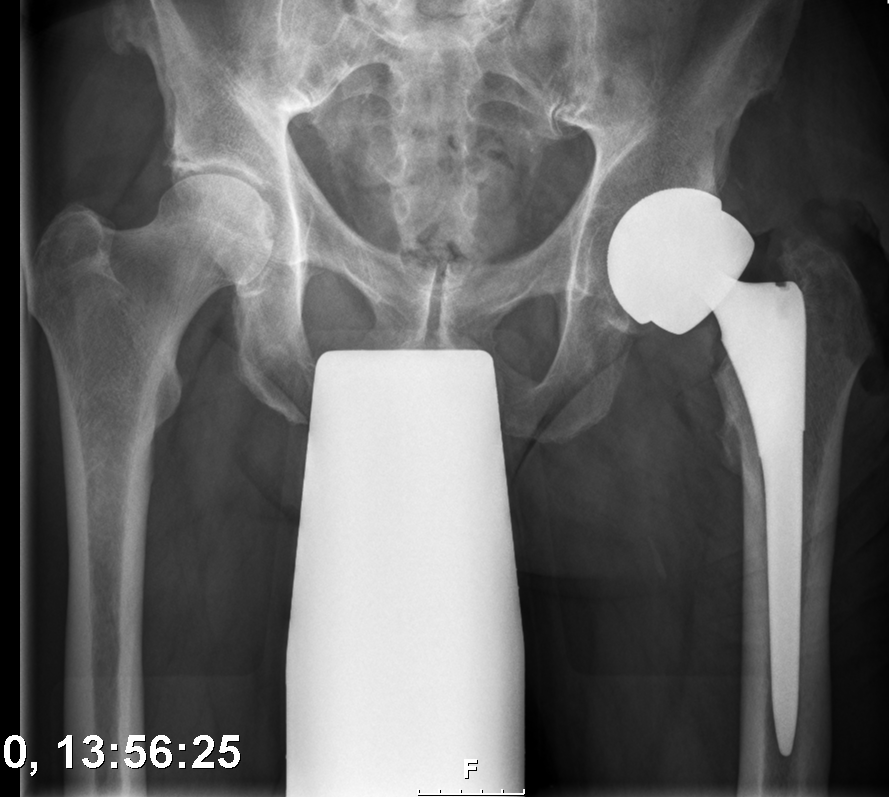 |  |
| 470- | 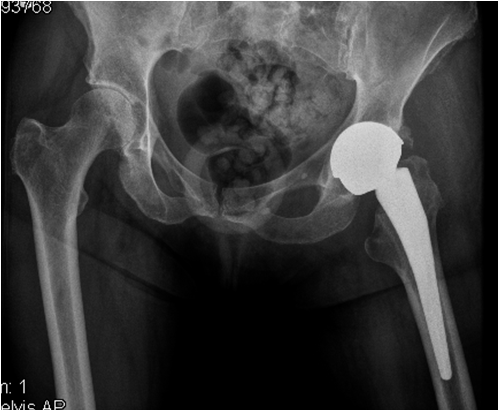 |  |
| 474-GM | 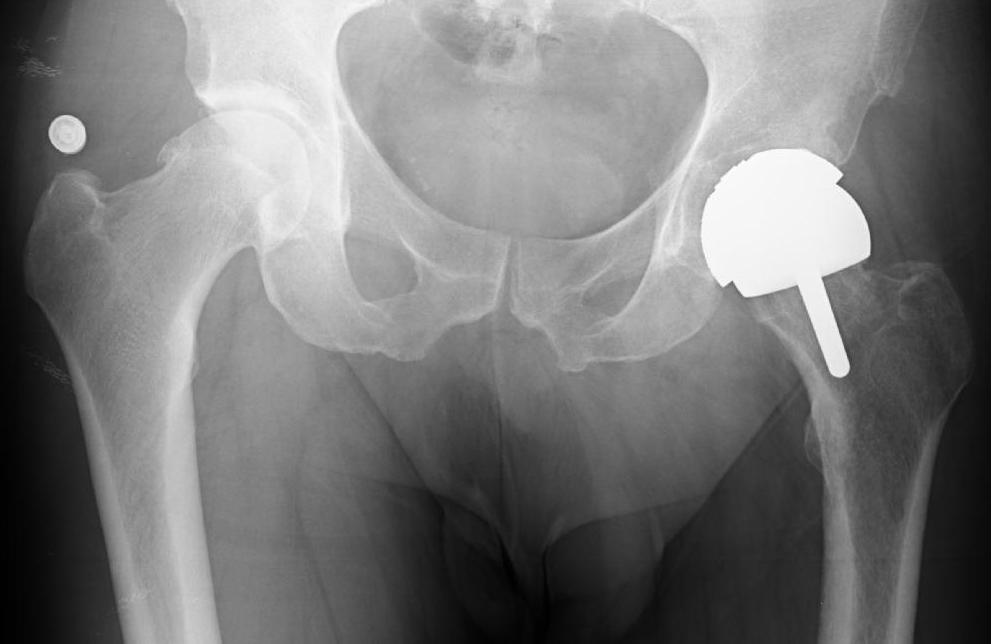 |  |
| 539-LM | 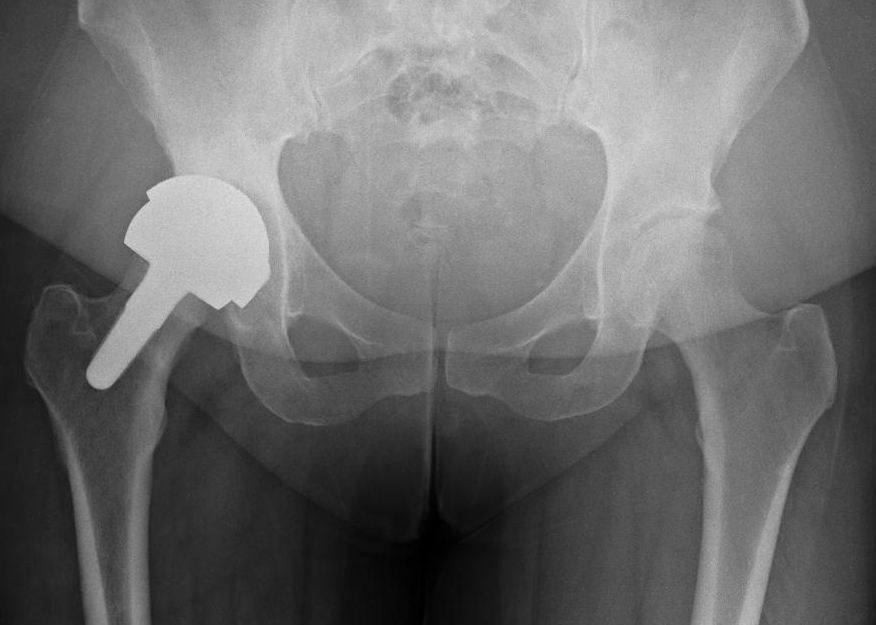 |  |
|  | | |
| 624-MB | 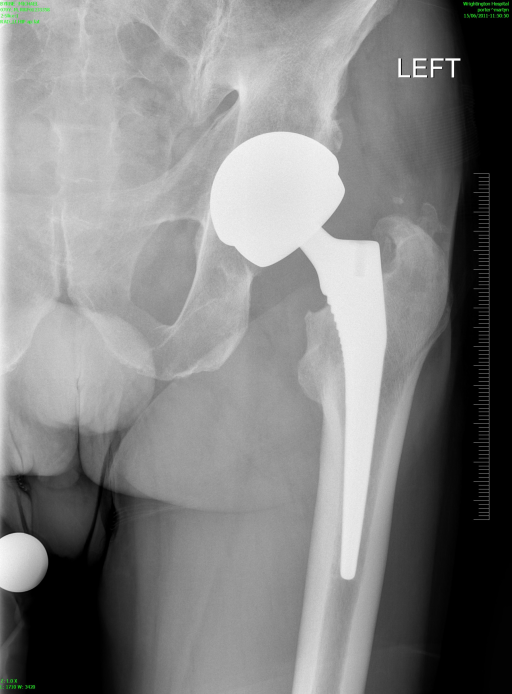 |  |
| 666-JC | 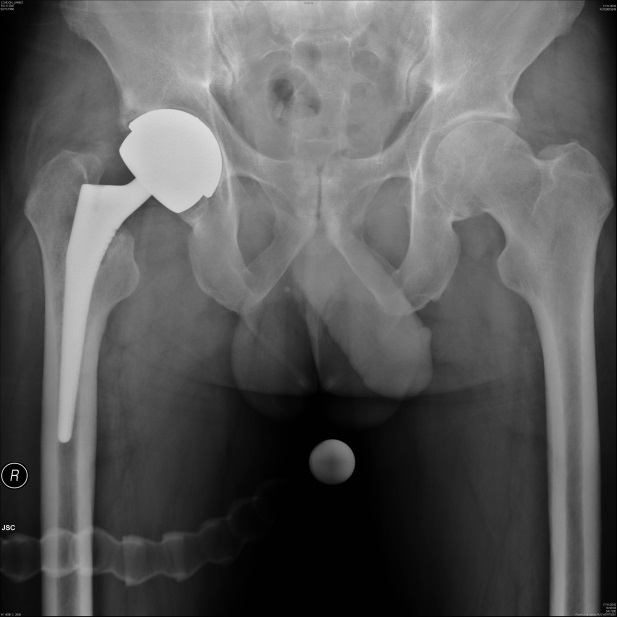 |  |
| 748-MK | 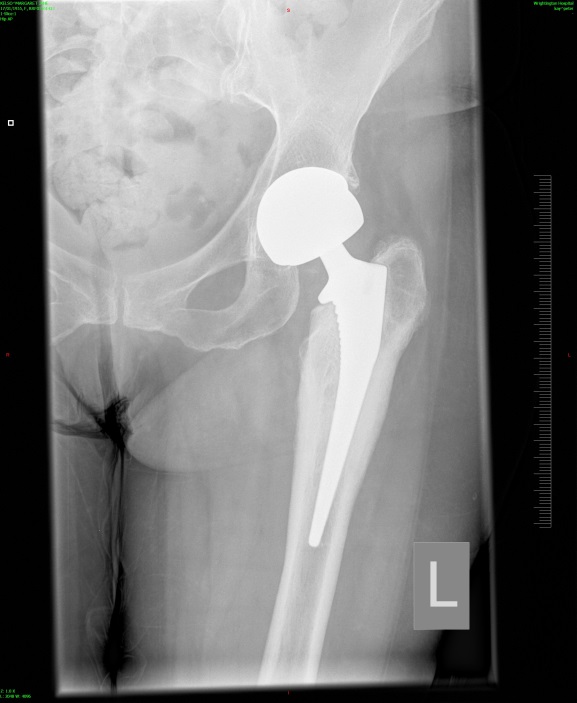 |  |
| 876-BK | 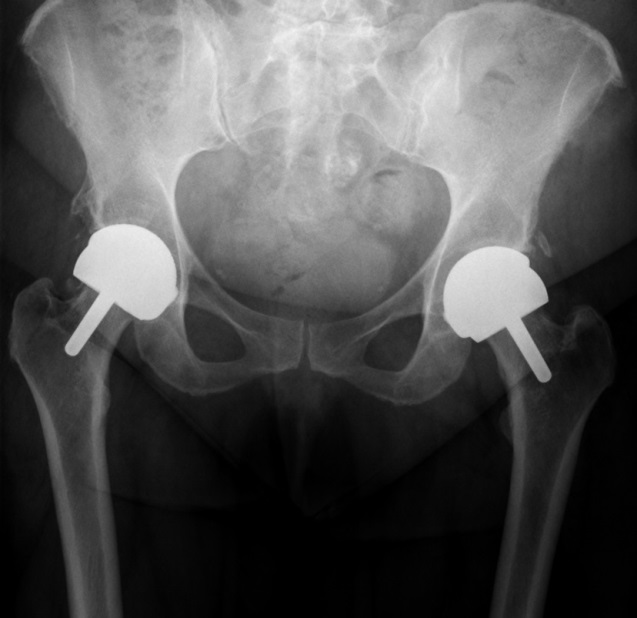 | (Please refer to Right Hip) |
| A03-PC | 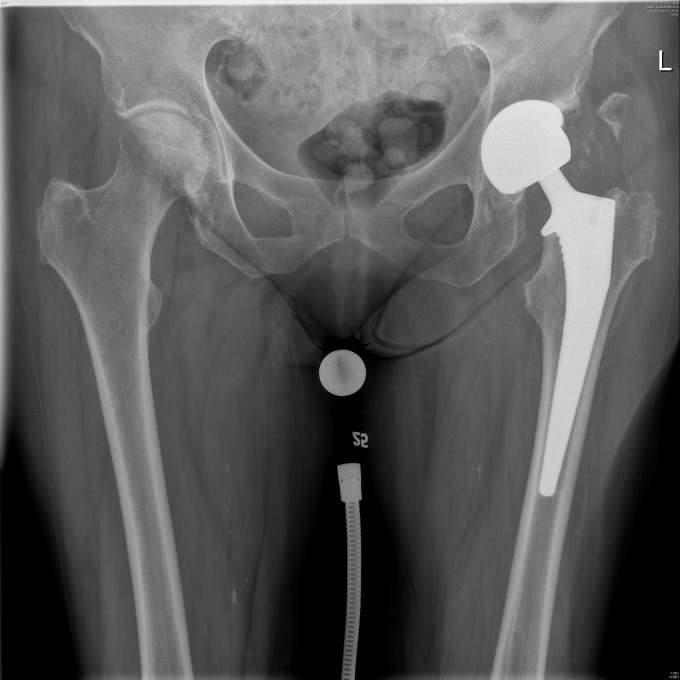 |  |
| A25-MD | 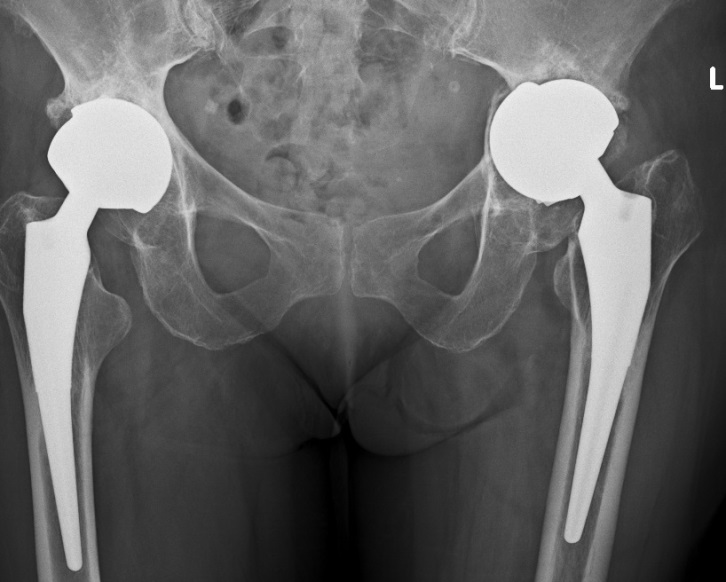 | (Please refer to Left Hip) |
| B07-LH | 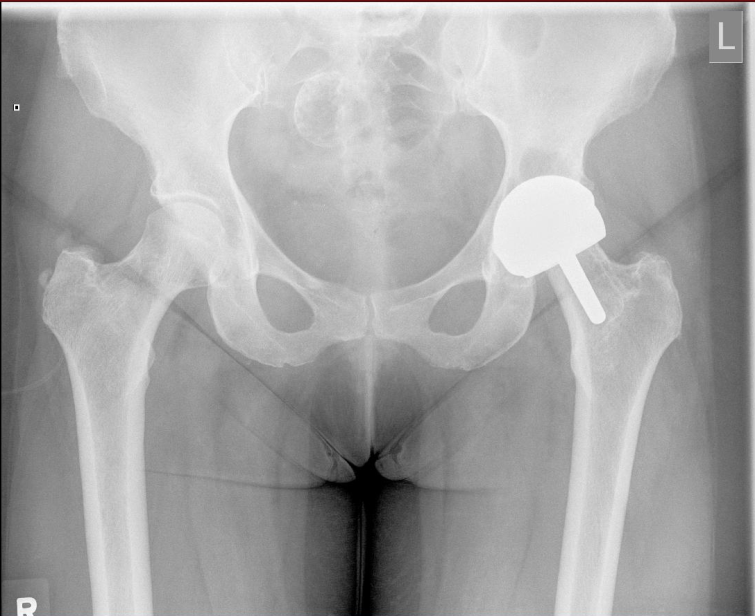 |  |
| 887-AP | 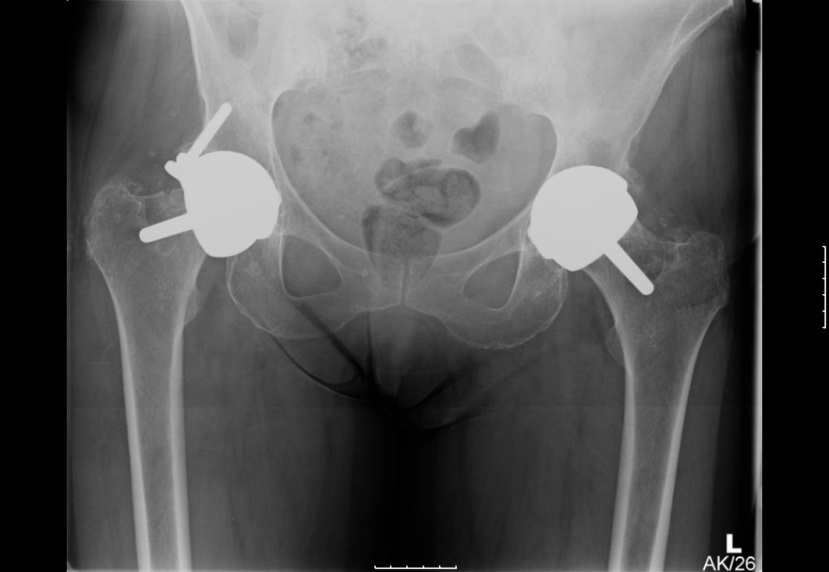 | (Please refer to Right Hip) |
| 145-GP | 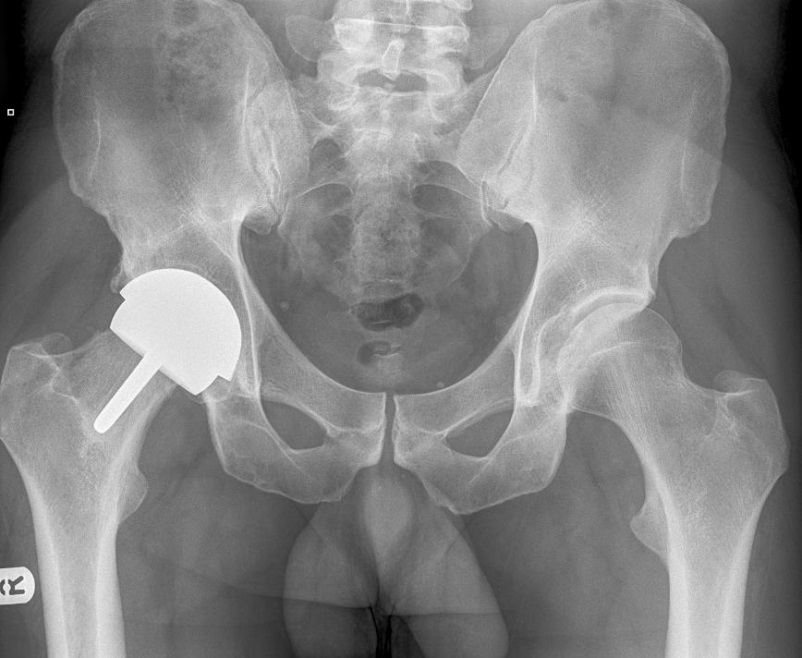 |  |
| 159-BC | 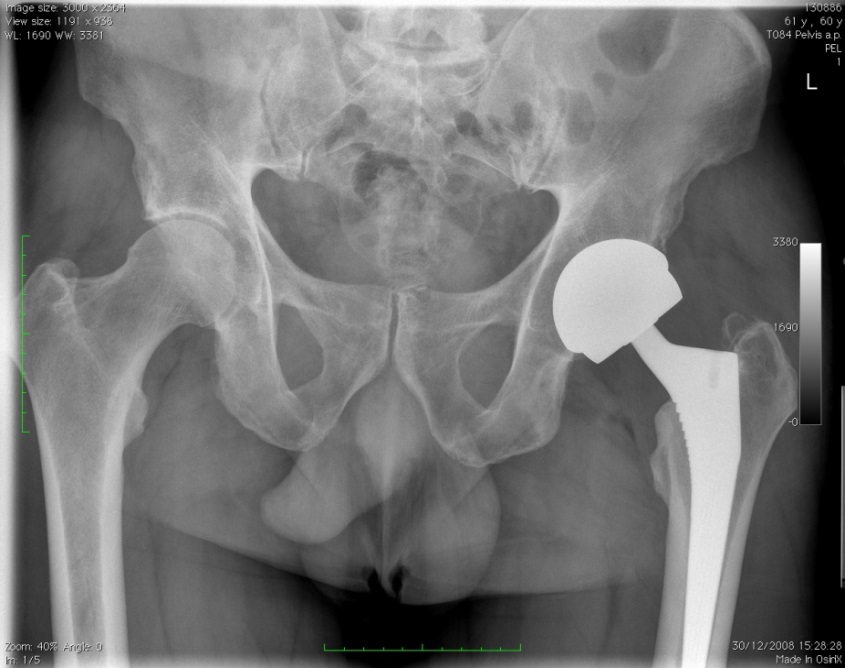 |  |

**Hip X-Ray Diagnoses – For Questionnaire**

019 – Aseptic Loosening of Cup

070 – Aseptic Loosening of Cup and Femoral Neck Lysis

159 – Osteolysis of Greater Trochanter and Acetabulum (Zone 1, 2 & 3)

229-DP – Septic Loosening Stem (Zones 1 & 7) and Cup with lysis of Acetbulum (Zones 1, 2 & 3)

107 – Calcar Resorption plus Acetabular Osteolysis (Zones 2 & 3)

176 – Aseptic Loosening/Lysis to stem Zones 1, 2, 6 & 7 (Left)

152 – AVN femoral head beneath resurfacing with Collapse into Varus position (Left)

145 – Aseptic Loosening of femoral component

287 – Osteolysis Greater Trochanter and Stem (Zones 1 and 7). (Left, NB Poor image quality)

310 – Superior Dislocation

311 – Keel/Stem breaching anterior femoral cortex

624 – Calcar Resorption

666 – Aseptic Femoral Loosening (Zones 1 & 7) and Cup (Zones 1 & 2)

748 – Possible infection / Changes to cortical bone morphology – Thickened cortices to stem

887 – Fracture NOF (Right, Peri-resurfacing stem)

A03 – Avulsion of Greater Trochanter

A25 – Cup migration with Fracture of medial Acetabulam. Osteolysis stem (Zones 1 & 7, Left)

B07 – Acetabular Osteolysis Zones 1 & 2

**Size Mismatch**

075 – Heterotopic Ossification, Acetbular Osteolysis (Zone 3)

121 – Aseptic Loosening Cup (Zones 2 & 3)

435 – Mismatch

470 – Mismatch

474 –Aseptic Loosening Stem

539 – Acetbular osteolysis (zones 2 & 3),

876 – Thinning of neck? (Right)
